# Supplementary material for: Machine-learning-assisted insight into spin ice Dy2Ti2O7
Source: Nat Commun. 2020 Feb 14;11:892. doi: 10.1038/s41467-020-14660-y (PMC7021707; doi:10.1038/s41467-020-14660-y)
Supplement: Supplementary file 1 — Supplementary Information [file 41467_2020_14660_MOESM1_ESM.pdf]

Supplementary Information: Machine-Learning-Assisted Insight into Spin Ice $Dy_2Ti_2O_7$

Anjana M Samarakoon*, Kipton Barros, Ying Wai Li, Markus Eisenbach, Qiang Zhang, Feng Ye, V. Sharma, Z. L. Dun, Haidong Zhou, Santiago A. Grigera, Cristian D. Batista, and D. Alan Tennant

This PDF file includes:

I. Additional information on experiment at CORELLI and data analysis
II. Heat Capacity data and simulation
III. Extra details on optimization and trained autoencoders
IV. Formulation of the optimal region

Supplementary Figures 1-7

Additional information on neutron experiment and data analysis

Background estimation for diffuse neutron scattering data

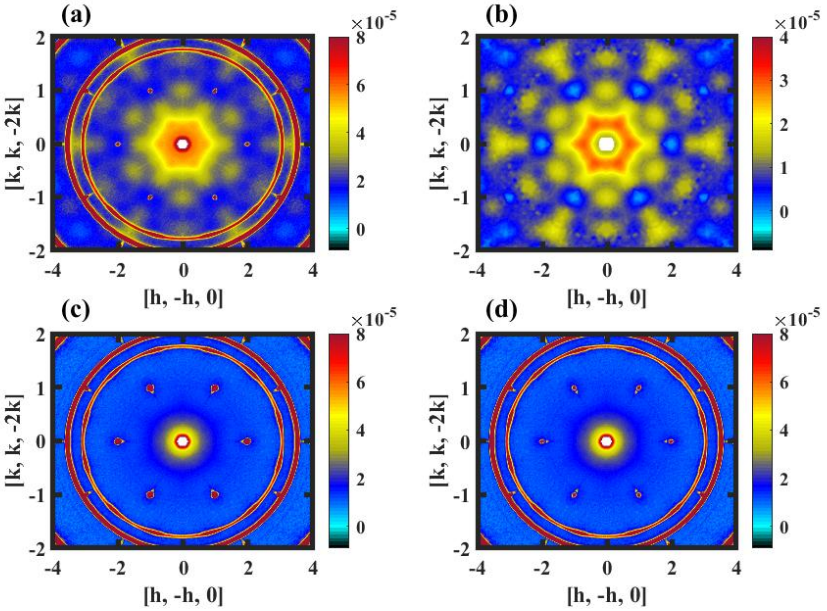

Figure 1: Background estimation for CORELLI data. Raw data from CORELLI experiment measured at 680 mK and magnetic field along [1,1,1] direction of 0 T (a) and 1.4 T (c) are shown here. The high-intensity rings are from the aluminum powder scattering from the sample holder and the instrument. The overall non-magnetic background (d) has been determined by replacing the Bragg peak intensity measured at 1.4 T by the zero-field values. The resulting background subtracted magnetic structure factor is shown in panel (b).

Supplementary Fig. 1 (a) and (c) shows the raw data for 680 mK experiment at 0T and 1.4 T respectively. Here the spins are fully polarized by the field of 1.4 T. This contributes magnetic signal at the Bragg peak positions *only*, which adds to the existing signal from the nuclear scattering from the crystal. Between Bragg peaks then represents non-magnetic background. The

zero-field data was used to estimate the non-magnetic Bragg scattering the 0 T data, where the magnetic contribution is expected to be zero (the net magnetization is equal to zero). The non-magnetic background then is estimated by replacing the Bragg peaks in the 1.4T data with those from the 0 T data to a width of 3σ, shown in the Supplementary Fig. 1 (d). This estimate of the background was used for all the 680 mK data sets covering different magnetic field values. An example of background subtracted data is shown in the Supplementary Fig. 1 (b) for the case of no field. In reality a small diffuse magnetic component is present at the 0 Tesla Bragg peak positions which is hard to deal with and results in over subtractions. An advantage of our machine learning approach is that it corrects for Bragg peaks by filtering them out rather than by subtraction, and allows diffuse magnetic signal to be accessed close to the peak positions. (see Fig. 2)

Comparison of data and simulation along different slices

The slices $[k, k, -2k] - [l, l, l]$ and $[h, -h, 0] - [l, l, l]$ of the background subtracted data and best model simulations are shown in the Supplementary Fig. 2.

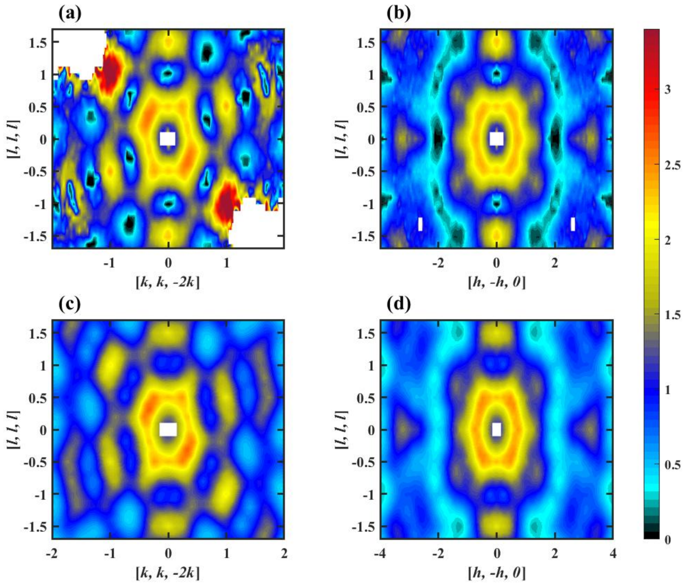

Figure 2: Comparison of the experimental and simulated data. The experimentally measured spin-spin correlations at 680mK (a)-(b) and modelling at the same temperature (c)-(d) along two perpendicular planes through $\mathbf{Q} = 0$ in reciprocal space as shown here.

Heat capacity data and simulation

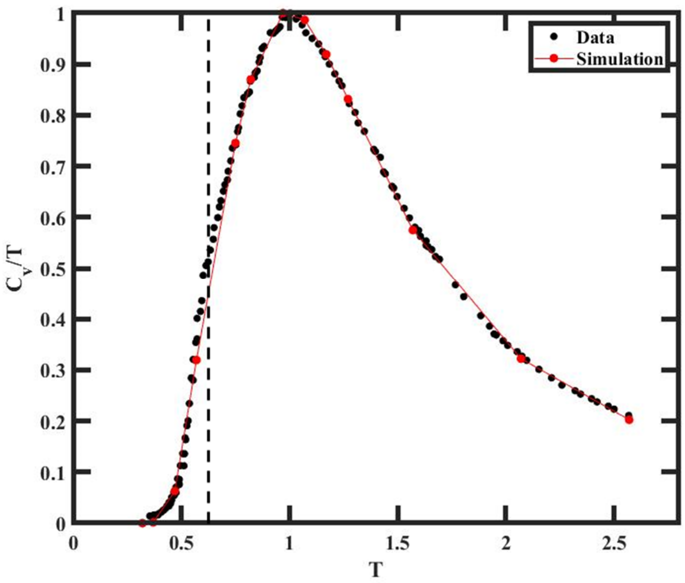

Figure 3: Comparison of specific heat data and calculation. Specific Heat Capacity (c_v) data from ref. [1] and simulation at the optimized parameters given in the main text as a function of renormalized temperature. Below 600 mK (black dashed line), the temperature dependence of c_v is controversial [2].

Extra details on optimization and trained autoencoders

Performance of Autoencoder filtering

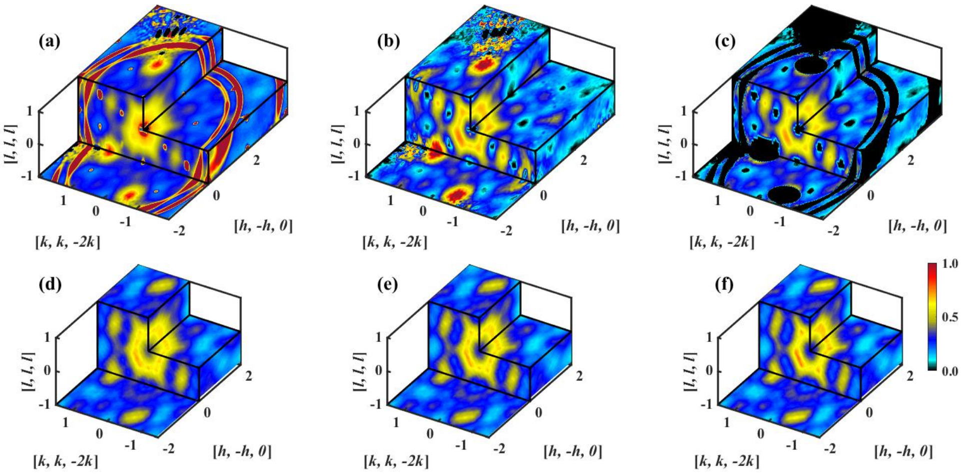

Figure 4: Autoencoder filtering on differently processed data. The filtration of (a) raw, (b) background subtracted and (c) masked data at 680 mK are compared. The instrumentation background was estimated as explained in Supplementary Fig. 1. The mask was applied to background-subtracted data so as to exclude all the proposed measurement artifacts. The masked data was used to calculated $\chi^2_{S(\mathbf{Q})}$ and χ^2_{AE} described in main text. (d), (e) and (f) are the autoencoder regularized structure factors, $S_{AE}(\mathbf{Q})$ for differently preprocessed input data (a), (b) and (c) respectively. Note that the axis limits of $S_{AE}(\mathbf{Q})$s are different from the original limits of $S^{exp}(\mathbf{Q})$ since the input datasets has been cropped to the same limits of $S^{sim}(\mathbf{Q})$ before regularizing through the trained autoencoder.

Extracted features by the autoencoder

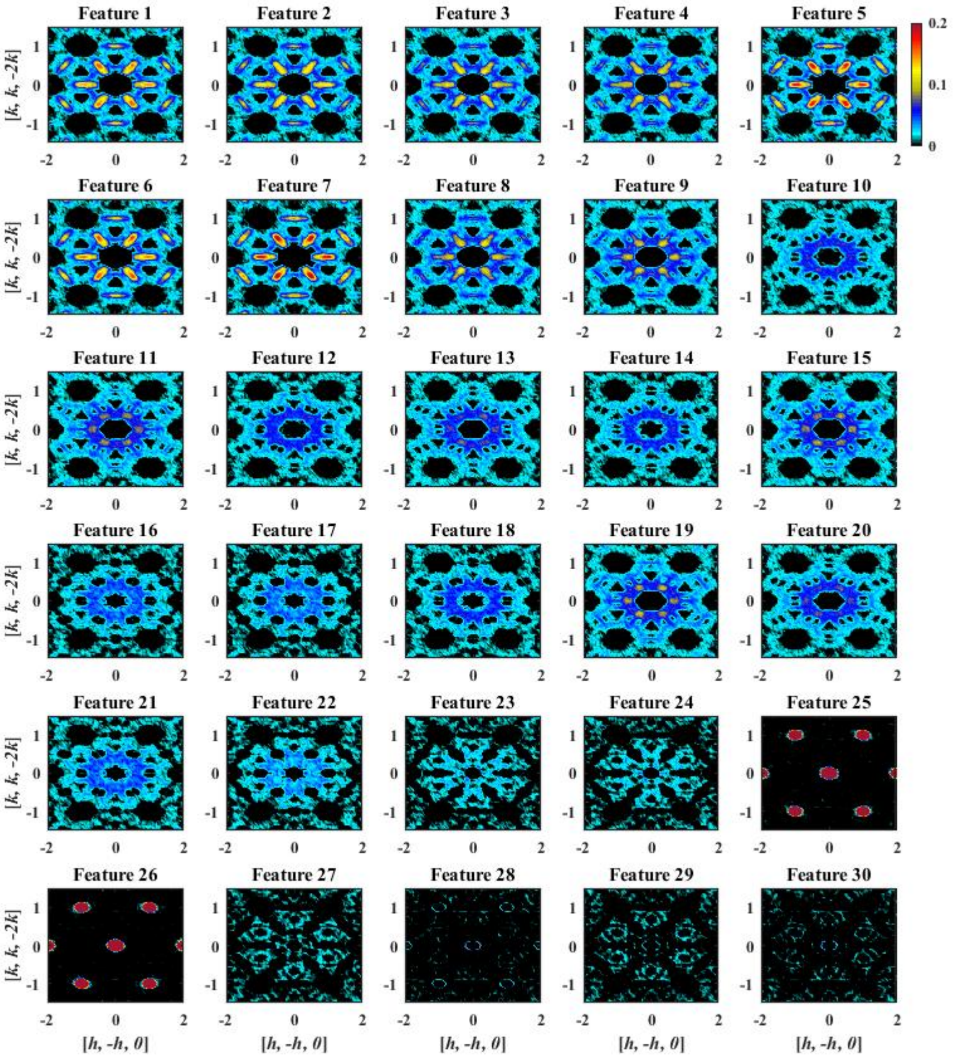

Figure 5: Extracted features by the autoencoder. The extracted 30 features of $S(\mathbf{Q})$ along $[h, -h, 0] - [k, k, -2k]$ plane from the autoencoder trained with 1000 random samples in the three-dimensional parameter space.

The activation of the latent space variables

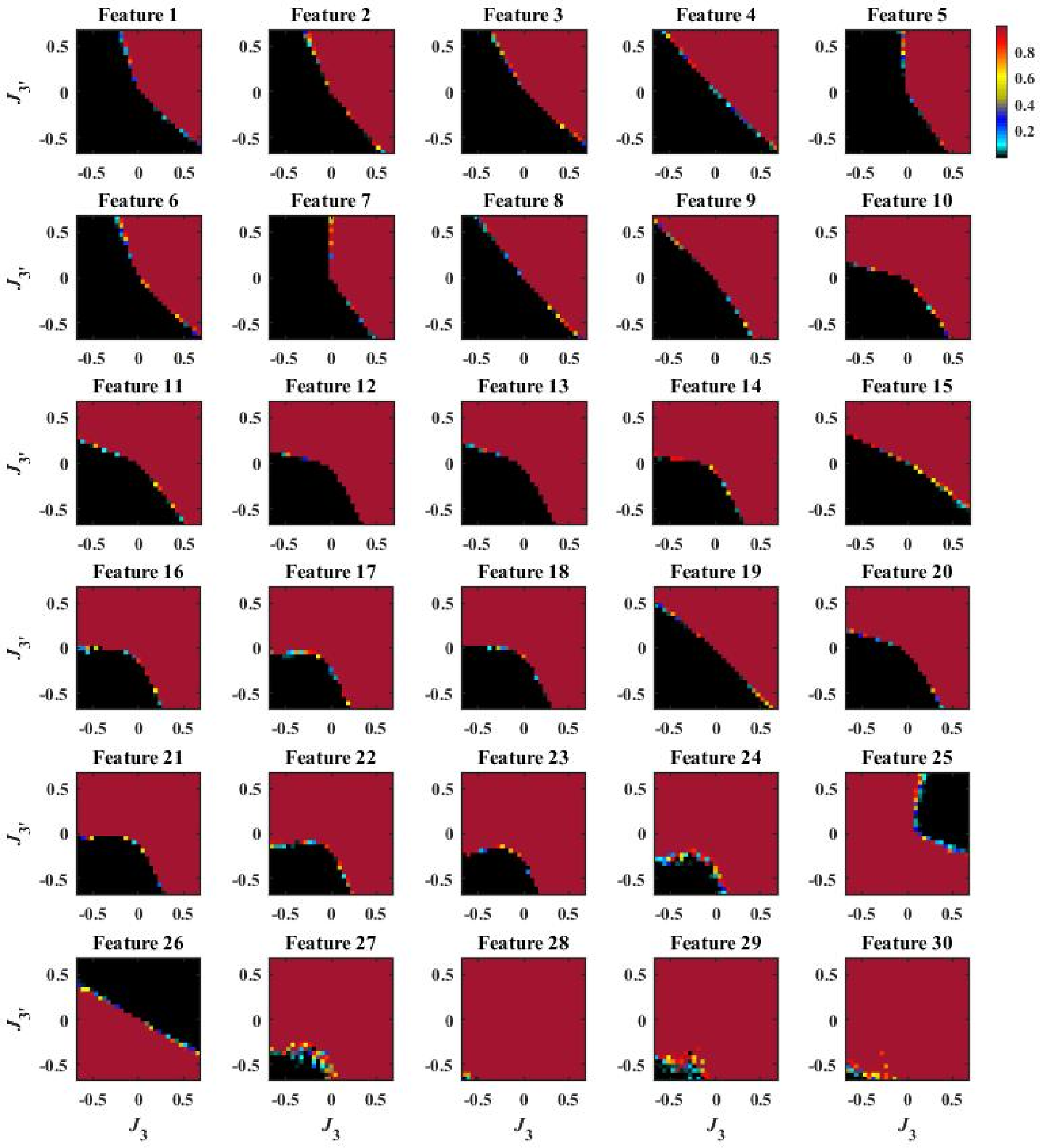

Figure 6: The activation of the latent space variables. The activation function along $J_3 - J_{3'}$ plane through $J_2 = 0$ for the 30 features shown on Supplementary Fig. 5.

Optimizing latent space dimension

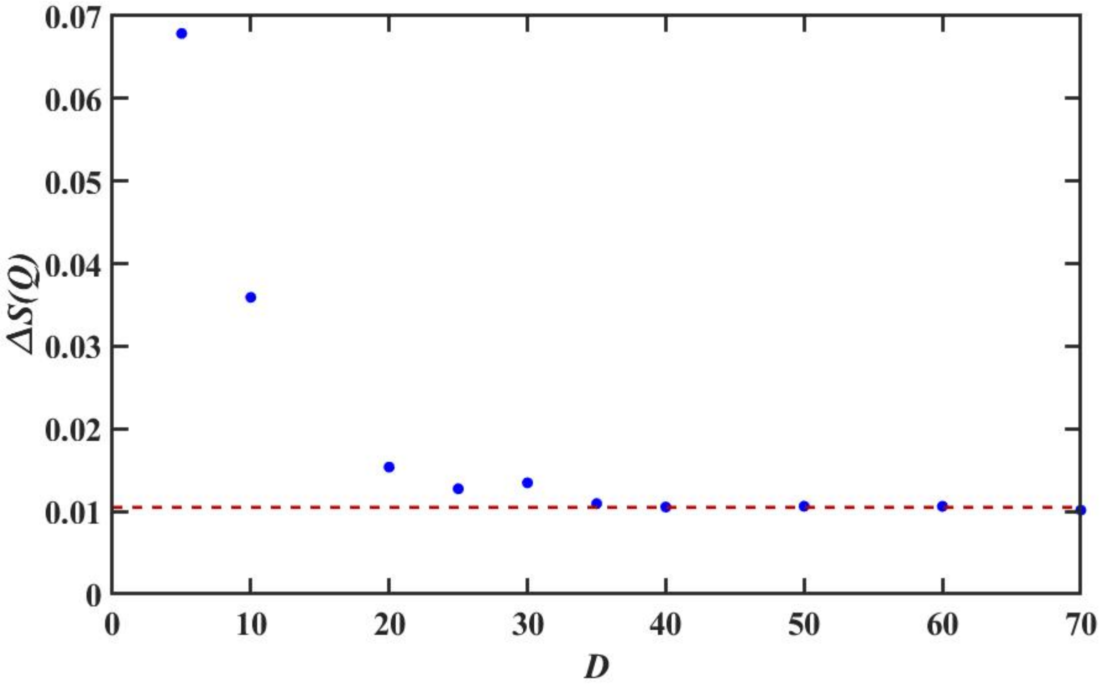

Figure 7: Optimizing latent space dimension. The total error over the validation data set, $\Delta S(\mathbf{Q})$ as a function of latent space dimension D.

Formulation of the optimal region

The three-dimensional optimal region for which $\chi^2_{\mathrm{multi}} < C^2_{\mathrm{multi}}$ as illustrated in Fig. 4(d) can as be formulated by fitting the region to a minimum volume ellipsoid [3] as,

$$V \times M \times V^{\dagger} \leq 1; \quad M = \begin{bmatrix} 3659 & -271 & 1046 \\ -271 & 3270 & 33 \\ 1046 & 33 & 322 \end{bmatrix} \quad V = [J_3 + 0.014 \quad J_{3'} - 0.102 \quad J_2 + 0.004], \ (1)$$

Note that, all the combinations of $J_2, J_3, J_{3'}$ which satisfies the above condition will reproduce both neutron structure factor and heat capacity behaviors.

The minimum volume episode for $\chi^2_{\mathrm{L}} < C^2_{\mathrm{L}}$ condition,

$$V \times M \times V^{\dagger} \leq 1; \quad M = \begin{bmatrix} 2834 & 487 & 948 \\ 487 & 969 & 167 \\ 948 & 167 & 320 \end{bmatrix} \quad V = [J_3 + 0.134 \quad J_{3'} - 0.102 \quad J_2 - 0.358], \ (2)$$

References

[1] Morris, D.J.P., Tennant, D.A., Grigera, S.A., Klemke, B., Castelnovo, C., Moessner, R., Czternasty, C., Meissner, M., Rule, K.C., Hoffmann, J.U. and Kiefer, K., "Dirac strings and magnetic monopoles in the spin ice Dy2Ti2O7.," Science, vol. 5951, no. 326, pp. 411-414, 2009.

[2] Pomaranski, D., L. R. Yaraskavitch, S. Meng, K. A. Ross, H. M. L. Noad, H. A. Dabkowska, B. D. Gaulin, and J. B. Kycia. , "Absence of Pauling's residual entropy in thermally equilibrated Dy 2 Ti 2 O 7.," Nature Physics, vol. 9, no. 6, p. 353, 2013.

[3] Moshtagh, Nima, "Minimum volume enclosing ellipsoid," Convex optimization, vol. 111, pp. 1-9, 2005.
